# EZH2-Mediated PTEN Silencing Promotes AKT-Dependent Afatinib Resistance in Radiation-Resistant Cervical Cancer Cells

**DOI:** 10.3390/jcm14207329

**Published:** 2025-10-17

**Authors:** Won-Hyoek Lee, Seong Cheol Kim, Sungchan Park, Jeong Woo Park, Sang-Hun Lee

**Affiliations:** 1 Biomedical Research Center, Ulsan University Hospital, University of Ulsan College of Medicine, Ulsan 44033, Republic of Korea; 0734955@uuh.ulsan.kr (S.C.K.); scpark@uuh.ulsan.kr (S.P.); 2 Department of Obstetrics & Gynecology, Ulsan University Hospital, University of Ulsan College of Medicine, Ulsan 44033, Republic of Korea; 3 Department of Urology, Ulsan University Hospital, University of Ulsan College of Medicine, Ulsan 44033, Republic of Korea; 4 School of Biological Sciences, University of Ulsan, Ulsan 44610, Republic of Korea; jwpark@ulsan.ac.kr

**Keywords:** cervical cancer, EZH2, PTEN, AKT pathway, afatinib, radioresistance, chemoresistance

## Abstract

**Highlights:**

**What are the main findings?**

- Radiation-acquired HeLaR cervical cancer cells exhibit significant afatinib resistance.
- EZH2 epigenetically silences PTEN, leading to sustained AKT activation.

**What is the implication of the main findings?**

- Pharmacologic inhibition of EZH2 or PI3K/AKT restores afatinib sensitivity.
- In vivo, combination therapy with an EZH2 inhibitor and afatinib suppresses tumor growth without toxicity.
- The EZH2–PTEN–AKT axis represents a potential therapeutic target in recurrent and radioresistant cervical cancer.

**Abstract:**

**Background**: Cervical cancer remains a major global health burden, and treatment failure due to radioresistance and secondary drug resistance severely limits clinical outcomes. Enhancer of zeste homolog 2 (EZH2) is a key epigenetic regulator implicated in tumor progression. This study aimed to determine whether EZH2-mediated PTEN silencing drives afatinib resistance via AKT activation in radiation-resistant cervical cancer cells. **Methods**: A radioresistant cervical cancer cell line (HeLaR) was established following cumulative irradiation (70 Gy). Cell viability, clonogenic survival, methylation-specific PCR (MSP), chromatin immunoprecipitation (ChIP), and Western blot analyses were conducted. EZH2 (Dznep; tazemetostat), PI3K, and AKT inhibitors were tested in combination with afatinib. A xenograft mouse model was used for in vivo validation. **Results**: HeLaR cells exhibited upregulation of EZH2 and H3K27me3, downregulation of PTEN, and sustained AKT activation. EZH2 inhibition restored PTEN expression, attenuated AKT phosphorylation, and re-sensitized cells to afatinib. MSP and ChIP confirmed EZH2-mediated PTEN promoter silencing. PI3K inhibition reproduced these effects, whereas ERK inhibition had minimal impact. In xenograft models, combined treatment with Dznep and afatinib significantly suppressed tumor growth compared to single agents. **Conclusions**: EZH2-driven PTEN suppression promotes AKT-dependent afatinib resistance in radiation-resistant cervical cancer. Targeting the EZH2–PTEN–AKT axis may provide a potential therapeutic approach to mitigate combined radioresistance and chemoresistance in recurrent cervical cancer, although further preclinical and clinical validation is required.

## 1. Introduction

Cervical cancer remains a major global health burden, ranking as the fourth most common malignancy among women worldwide [1]. Radiotherapy is an essential treatment modality, particularly for locally advanced disease [2]; however, the emergence of resistance to radiation reduces therapeutic efficacy and increases recurrence rates. Moreover, tumors that survive irradiation often display cross-resistance to structurally and mechanistically distinct anticancer drugs, further complicating treatment strategies [3,4].

The epidermal growth factor receptor (EGFR) pathway plays a pivotal role in tumor proliferation, survival, and therapy evasion [5]. Upon activation, EGFR triggers downstream cascades such as the phosphoinositide 3-kinase (PI3K)/AKT and mitogen-activated protein kinase (MAPK)/extracellular signal-regulated kinase (ERK) pathways, both of which are key mediators of oncogenesis and treatment failure [6,7].

Despite the pivotal role of EGFR signaling, current international guidelines (ESGO, NCCN, ASCO, FIGO, ESMO, and others) for recurrent cervical cancer primarily recommend platinum-based chemotherapy, bevacizumab, and immunotherapy, without incorporating EGFR inhibitors [8]. This underscores the need to explore novel strategies, such as targeting the EGFR–EZH2–PTEN–AKT axis, to overcome therapeutic resistance.

Beyond signaling dysregulation, epigenetic alterations contribute to therapy adaptation. Enhancer of zeste homolog 2 (EZH2), the catalytic subunit of the polycomb repressive complex 2 (PRC2), silences gene transcription via histone H3 lysine 27 trimethylation (H3K27me3) [9]. EZH2 overexpression has been linked to aggressive phenotypes and poor prognosis across multiple cancers [10,11].

The tumor suppressor phosphatase and tensin homolog (PTEN) negatively regulates PI3K/AKT signaling, thereby constraining tumor growth and survival [12]. Epigenetic silencing of PTEN via promoter methylation results in constitutive AKT activation and has been implicated in drug resistance [13]. Whether EZH2-driven PTEN suppression contributes to therapy failure in the context of radiation-acquired adaptation in cervical cancer remains unclear.

In this study, we established a radioresistant cervical cancer cell line (HeLaR) by exposing parental HeLa cells to a cumulative dose of 70 Gy. We investigated whether EZH2-mediated epigenetic regulation contributes to afatinib insensitivity and whether pharmacologic inhibition of EZH2 could restore PTEN function, suppress AKT signaling, and re-sensitize HeLaR cells to EGFR-targeted therapy, both in vitro and in vivo.

## 2. Materials and Methods

### 2.1. Study Design

This study was designed to evaluate whether EZH2-mediated PTEN silencing is involved in the acquisition of afatinib resistance in radioresistant cervical cancer cells. Radioresistant HeLa cells (HeLaR) were generated by fractionated irradiation (cumulative 70 Gy). Subsequent in vitro experiments included viability assays, Western blotting, methylation-specific PCR (MSP), and chromatin immunoprecipitation (ChIP). Therapeutic relevance was assessed in xenograft mouse models. All procedures were conducted under approved institutional protocols.

### 2.2. Reagents

Afatinib (provided by the pharmacy unit of Ulsan University Hospital, manufactured by Sanofi-Aventis, Bridgewater, NJ, USA), Dznep, tazemetostat, LY294002, PD98059, MK-2206 (Selleckchem, Houston, TX, USA) were used at indicated concentrations.

### 2.3. Ethics Statement

This study was approved by the Institutional Review Board (IRB) and ethics committee of Ulsan University Hospital (IRB number: NON2020-003; approval date: 28 August 2020). All animal experiments were performed in accordance with procedures approved by Institute of Laboratory Animal Resources, University of Ulsan (Ethical code number: 032-01 [A1-0]).

### 2.4. Cell Culture and Establishment of Radioresistant Cervical Cancer Cell Line

HeLa cell lines were purchased from the Korean cell line bank (Seoul, Republic of Korea). The HeLa cell lines were cultured in RPMI-1640 (WELGENE, Gyeongsan, Republic of Korea), each supplemented with 10% fetal bovine serum (Invitrogen, Carlsbad, CA, USA) and 1% penicillin/streptomycin (Invitrogen). Cells were cultured at 37 °C in a humidified chamber containing 5% carbon dioxide. HeLa cells were grown to approximately 50% confluence in vented 75-cm^2^ culture flasks and irradiated using 6-MV photon beam generated by a linear accelerator (CLINAC 600; Varian, Palo Alto, CA, USA) at a dose rate of 2 Gy. After the cells received a cumulative dose of 70 Gy, radioresistant cells were designated as HeLaR.

### 2.5. Viability Assay

At the indicated times, the CellTiter 96^®^ Aqueous One Solution Reagent (Promega, Madison, WI, USA) was added to each well according to the manufacturer’s instructions. Then, absorbance at 490 nm (OD490) was determined for each well using the Wallac Vector 1420 Multilabel Counter (EG&G Wallac, Turku, Finland).

### 2.6. Western Blot Analysis

Total protein was extracted using a RIPA buffer containing proteases and phosphatase inhibitors (Thermo Fisher Scientific, Waltham, MA, USA), and protein concentration was determined using the Bradford protein assay kit (Bio-Rad Laboratories, Hercules, CA, USA). Proteins were separated by electrophoresis on a 10–13% SDS polyacrylamide gel, and transferred to a nitrocellulose membrane (Amersham International, Little Chalfont, UK). Membranes were blocked with 5% bovine serum albumin (BSA; bioWORLD, Dublin, OH, USA) in Tris-buffered saline with tween 20 (TBST) for 1 h at room temperature. Membranes were subsequently washed with TBST and incubated with primary antibodies to EZH2 (ab3748, Abcam, Cambridge, UK), H3K27me3 (ab6002, Abcam), ERK (#9102, Cell Signaling, Danvers, MA, USA), phospho-ERK (#9101, Cell Signaling), EGFR (#4267, Cell Signaling), PI3K p110 alpha (#4249, Cell Signaling), PI3K p85 (#4292, Cell Signaling), PI3K phospho-p85 (#4228, Cell Signaling), AKT (sc-5298, Santa Cruz Biotechnology, Santa Cruz, CA, USA), phospho-AKT (Ser473) (sc-7985-R, Santa Cruz Biotechnology), PTEN (sc-7974, Santa Cruz Biotechnology) and β-actin (sc-47778, Santa Cruz Biotechnology) and diluted in 5% BSA/TBST overnight at 4 °C. Membranes were washed with TBST. The secondary antibody (anti-mouse or anti-rabbit immunoglobulin (Ig)-G HRP conjugate; Bethyl Laboratories, Montgomery, TX, USA) was diluted 2000-fold in TBST and applied to cells for 1 h. After washing the cells with TBST, the specific binding of antibodies was detected using an ECL kit (Thermo Fisher Scientific), following the manufacturer’s protocol.

### 2.7. Methylation-Specific Polymerase Chain Reaction for PTEN

Genomic DNA was isolated from cell lines using standard procedures. One microgram of genomic DNA was treated with sodium bisulfite using the EpiTect Bisulfite kit (Qiagen, Valencia, CA, USA). This treatment converts all unmethylated cytosines into uracil. In the subsequent methylation-specific polymerase chain reaction (MSP), all of the uracils become thymidines. Polymerase chain reaction (PCR) requires primer pairs that specifically recognize methylated or unmethylated sequences. These primers were designed in the 5′ untranslated region CpG island of the published sequences.

The primer sequences were as follows (5′- to -3′): PTEN, GTGTTGGTGGAGGTAGTTGTTT (unmethylated sense), TTCGTTCGTCGTCGTCGTATTT (methylated sense), ACCACTTAACTCTAAACCACAACCA (unmethylated antisense), and GCCGCTTAACTCTAAACCGCAACCG (methylated antisense). PCR amplification was performed using the EpiTect MSP kit (Qiagen). Thermal cycling conditions were as follows: 1 cycle at 95 °C for 10 min; 35 cycles at 94 °C for 15 s, 60 °C for 1 min, and 72 °C for 30 s; and 1 cycle at 72 °C for 10 min. Each PCR (10 μL) was directly loaded onto 2% agarose gels stained with ethidium bromide (0.5 μg/mL) and directly visualized under ultraviolet light.

### 2.8. Chromatin Immunoprecipitation Assays

Chromatin immunoprecipitation (ChIP) assays were performed using the Pierce™ Agarose ChIP Kit (Thermo Fisher Scientific, Waltham, MA, USA), following the manufacturer’s instructions. After the samples were harvested and washed twice with PBS, chromatin was cross-linked with 1% formaldehyde for 10 min at room temperature. Next, 1.25 M glycine was added to quench the excess formaldehyde. The cells were pelleted by centrifugation, resuspended in lysis buffer, and sonicated to generate 200 to 500 bp DNA fragments using a Bioruptor sonicator (Diagenode Inc., Sparta, NJ, USA). The diluted chromatin solution was immunoprecipitated with 2 µg of anti-EZH2 (Cell signaling, Danvers, MA, USA) or rabbit immunoglobulin G. After washing, cross-link reversal, DNA elution, and DNA purification, the relative amount of immunoprecipitated DNA was quantified via qPCR using the primers.

### 2.9. Antitumor Activity in the Xenograft Model

HeLaR cells were injected into the flank of 6-week-old nude (nu/nu) mice (Orient Bio, Gyeonggi-do, Republic of Korea). Prior to treatment with Dznep and Afatinib, the tumor size was measured two to three times per week until the volume reached approximately 100 mm^3^. The tumor volume was calculated as W2 × L × 0.52, where L is the largest diameter and W is the diameter perpendicular to L. After establishment of these tumor xenografts, mice were randomized into four groups of five mice per group. Mice were fed ad libitum and maintained in environments with a controlled temperature of 22–24 °C and 12-h light and dark cycles. Dznep (2 mg/kg) and afatinib (12.5 mg/kg) were administered intratumorally twice per week for two weeks, and tumor growth was subsequently monitored for a total of three weeks.

### 2.10. Statistical Analysis

Data are expressed as mean ± standard deviation (SD). Statistical analyses were performed using GraphPad Prism 9.0 (GraphPad Software, San Diego, CA, USA). For comparisons between two groups, Student’s *t*-test was used. For experiments involving more than two groups, one-way ANOVA followed by Tukey’s post hoc multiple comparison test was applied. A *p*-value < 0.05 was considered statistically significant.

## 3. Results

### 3.1. Establishment of the HeLaR Cell Line and Confirmation of Radioresistance

**Figure 1:**
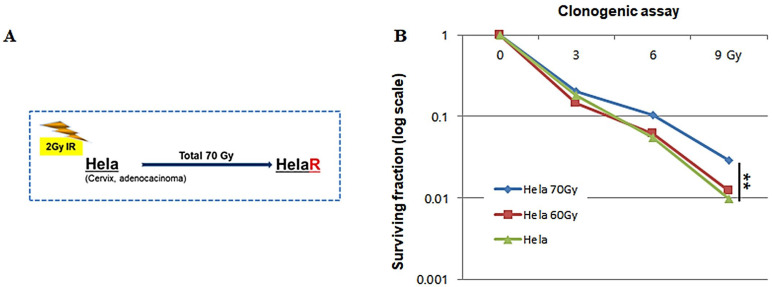
Establishment of the HeLaR cell line and confirmation of radiation adaptation. (**A**) Workflow illustrating generation of HeLaR cells from parental HeLa via repeated irradiation (2–4 Gy per fraction; cumulative 70 Gy). (**B**) Clonogenic survival after irradiation (0–9 Gy). HeLaR shows higher survival fractions than HeLa at 6 Gy and 9 Gy (** *p* < 0.01). Statistical analysis was performed as described in the Methods section.

HeLa cells subjected to a cumulative dose of 70 Gy irradiation successfully yielded a radioresistant HeLaR cell line (Figure 1A). Clonogenic assays demonstrated significantly higher survival of HeLaR cells compared to parental HeLa cells at 6 Gy and 9 Gy (Figure 1B). The resulting isogenic HeLaR population exhibited stable growth characteristics and was used for subsequent experiments.

### 3.2. Alterations in EGFR Signaling and Reduced Sensitivity to Afatinib in HeLaR Cells

**Figure 2:**
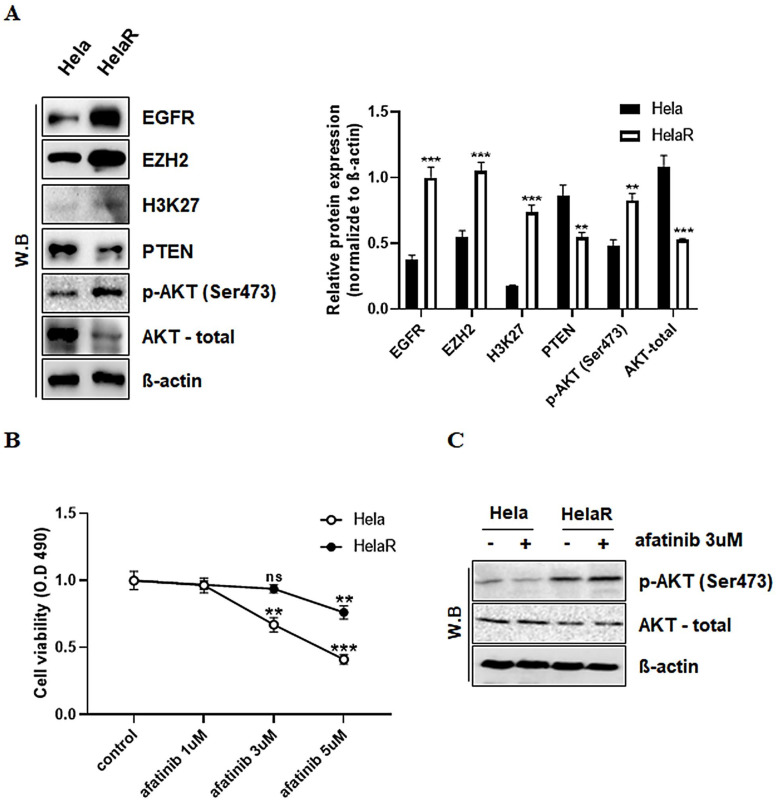
EGFR signaling alterations and reduced sensitivity to afatinib in HeLaR cells. (**A**) Western blots showing increased EGFR, EZH2, and H3K27me3, reduced PTEN, and elevated p-AKT in HeLaR vs. HeLa. (**B**) Line graph of cell viability (MTS) after afatinib treatment at 1, 3, or 5 µM for 24 h. (**C**) Western blots for p-AKT (Ser473) following afatinib treatment in HeLa and HeLaR cells. Data are expressed as mean ± SD from three independent experiments. Statistical analysis was performed as described in the Methods section. *p* < 0.05 (**), *p* < 0.01 (***) vs. control.

To explore molecular differences associated with radioresistance, we compared the expression of key oncogenic and regulatory proteins between HeLa and HeLaR cells. Western blotting revealed a marked increase in EGFR and its downstream effector p-AKT (Ser473) in HeLaR cells, along with elevated expression of the histone methyltransferase EZH2 and its target histone mark H3K27me3. In contrast, the tumor suppressor PTEN, a known negative regulator of AKT signaling, was downregulated in HeLaR cells (Figure 2A). These changes suggested a possible link between EZH2-mediated epigenetic regulation and suppression of PTEN, leading to hyperactivation of AKT. We next examined whether these molecular alterations influenced the response to afatinib, an EGFR-targeting tyrosine kinase inhibitor. Cell viability assays demonstrated that HeLaR cells retained significantly higher survival rates than HeLa cells across a range of afatinib concentrations (1–5 µM), indicating the acquisition of afatinib resistance (Figure 2B). To further validate AKT pathway involvement, we assessed phosphorylation at both Ser473 following afatinib treatment. Western blot analysis showed that afatinib effectively decreased p-AKT levels in HeLa cells, whereas HeLaR cells maintained elevated phosphorylation despite treatment, confirming persistent AKT activation in the resistant phenotype (Figure 2C).

### 3.3. Reversal of Afatinib Resistance by EZH2 Inhibition

**Figure 3:**
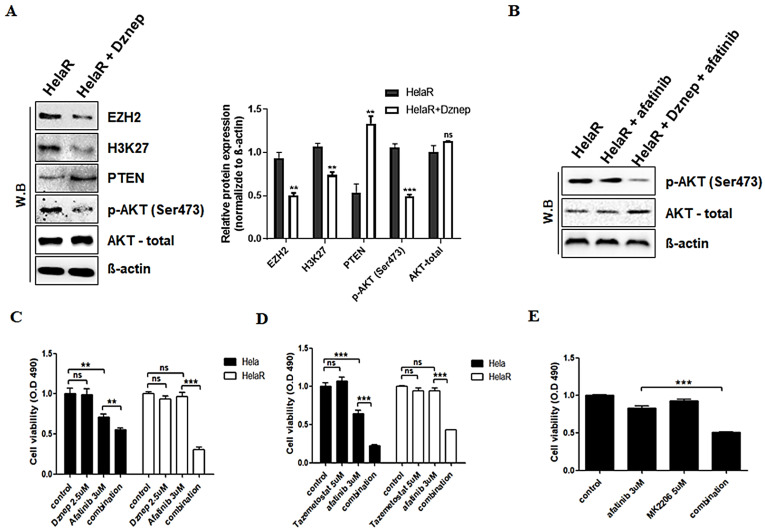
EZH2 inhibition reverses afatinib insensitivity. (**A**) HeLaR treated with Dznep (2.5 µM, 24 h) shows reduced EZH2/H3K27me3, restored PTEN, and decreased p-AKT. (**B**) Western blots showing reduced p-AKT with Dznep + afatinib vs. afatinib alone in HeLaR. (**C**,**D**) Viability (MTS) after Dznep (2.5 µM, 24 h) or tazemetostat (5 µM, 24 h) with/without afatinib (3 µM, 24 h). (**E**) AKT inhibitor MK2206 (5 µM, 24 h) mimics EZH2 blockade. Data are expressed as mean ± SD from three independent experiments. Statistical analysis was performed as described in the Methods section. *p* < 0.05 (**), *p* < 0.01 (***) vs. control.

Given the overexpression of EZH2 in HeLaR cells, we tested whether pharmacological inhibition of EZH2 could restore PTEN function and re-sensitize cells to afatinib. Treatment with the EZH2 inhibitor Dznep (2.5 µM, 24 h) reduced EZH2 and H3K27me3 levels, restored PTEN expression, and decreased p-AKT (Ser473) levels (Figure 3A). We next examined whether combination treatment more effectively suppressed AKT signaling. Western blot analysis showed that afatinib alone had only a limited effect on reducing p-AKT in HeLaR cells, whereas the combination of Dznep and afatinib markedly suppressed AKT phosphorylation (Figure 3B). In cell viability assays, both Dznep and the clinically approved EZH2 inhibitor tazemetostat (5 µM, 24 h) significantly enhanced the sensitivity of HeLaR cells to afatinib (Figure 3C,D). Similar effects were observed with direct AKT inhibition using MK2206 (5 µM, 24 h) (Figure 3E), confirming that excessive AKT activation downstream of EZH2–PTEN regulation plays a central role in afatinib resistance.

### 3.4. Role of PI3K and ERK Pathways in HeLaR Cell Survival

**Figure 4:**
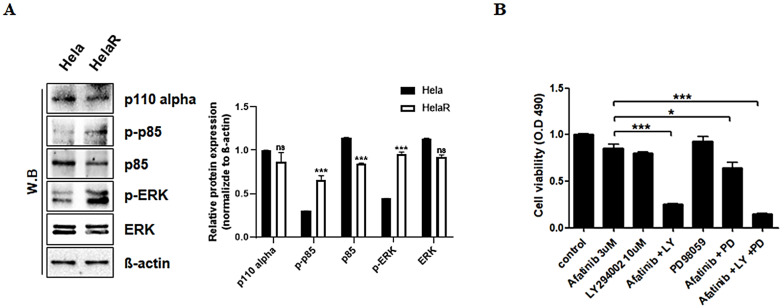
Role of PI3K and ERK pathways. (**A**) Western blots showing constitutive p-PI3K p85 and p-ERK in HeLaR. (**B**) Viability (MTS) after afatinib (3 µM, 24 h) combined with LY294002 (10 µM, 24 h) or PD98059 (10 µM, 24 h). Data are expressed as mean ± SD from three independent experiments. Statistical analysis was performed as described in the Methods section. *p* < 0.05 (*), *p* < 0.01 (***) vs. control.

To dissect the signaling cascades contributing to afatinib resistance, we analyzed two major EGFR downstream pathways: PI3K/AKT and MAPK/ERK. Western blotting showed constitutive phosphorylation of the PI3K p85 subunit and ERK in HeLaR cells (Figure 4A). Combination treatment with afatinib and the PI3K inhibitor LY294002 (10 µM, 24 h) significantly reduced cell viability, producing effects similar to EZH2 inhibition. In contrast, afatinib combined with the ERK inhibitor PD98059 (10 µM, 24 h) did not significantly affect survival (Figure 4B), indicating that AKT signaling plays the predominant role in mediating drug resistance in HeLaR cells.

### 3.5. Epigenetic Silencing of PTEN by EZH2

**Figure 5:**
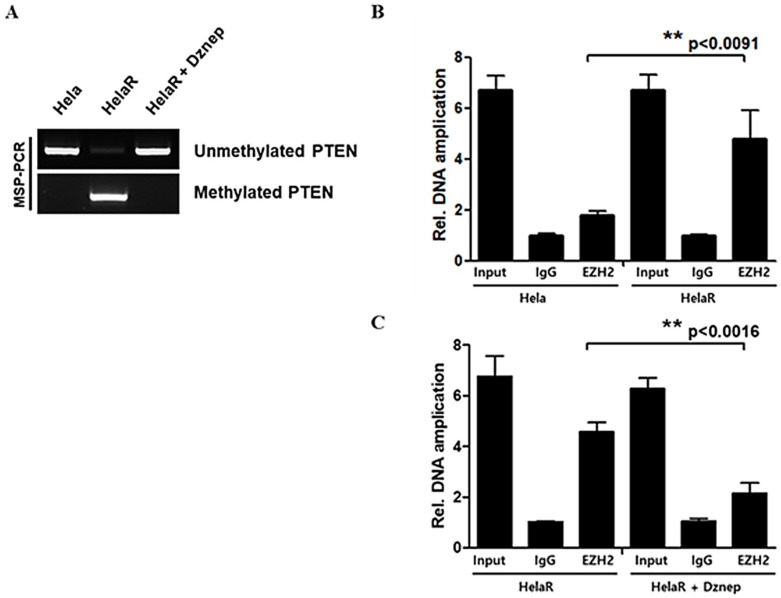
Epigenetic silencing of PTEN by EZH2. (**A**) MSP reveals a methylated PTEN promoter in HeLaR but not in HeLa; Dznep treatment shifts toward the unmethylated state. (**B**,**C**) ChIP showing higher EZH2 occupancy at the PTEN promoter in HeLaR vs. HeLa, reduced after Dznep. Quantification data are presented as mean ± SD from three independent experiments. Statistical significance was analyzed using one-way ANOVA followed by Tukey’s post hoc test. *p* < 0.05 (**).

To determine whether EZH2 suppresses PTEN via promoter methylation, we performed methylation-specific PCR (MSP). HeLaR cells displayed a methylated PTEN promoter, whereas parental HeLa cells showed an unmethylated profile. Treatment with Dznep induced a shift to the unmethylated state, restoring PTEN expression (Figure 5A). Chromatin immunoprecipitation (ChIP) assays further demonstrated increased EZH2 binding to the PTEN promoter in HeLaR cells compared to HeLa cells, with significantly reduced binding after Dznep treatment (Figure 5B,C). These findings confirm that EZH2 directly represses PTEN transcription through promoter methylation in the context of radioresistance.

### 3.6. In Vivo Validation in Xenograft Models

**Figure 6:**
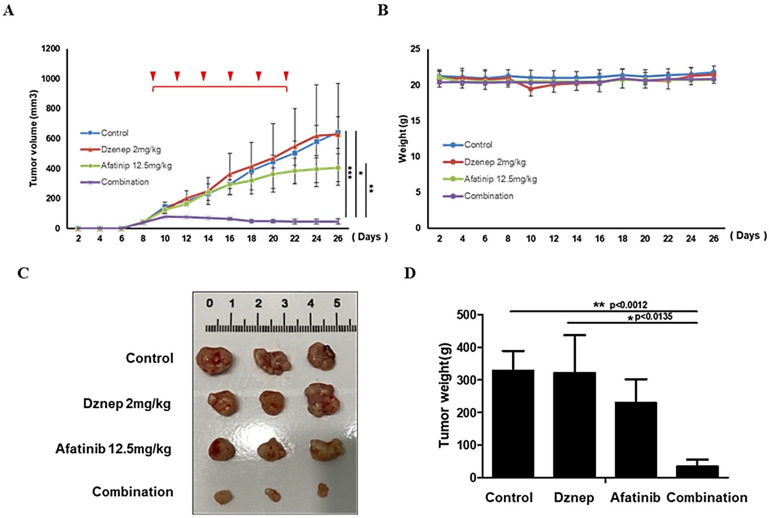
In vivo validation in xenograft models. HeLaR xenografts were treated intratumorally with Dznep (2 mg/kg, twice weekly) and/or afatinib (12.5 mg/kg, twice weekly) for two weeks, and tumor growth was subsequently monitored for a total of three weeks. (**A**) Tumor growth curves; (**B**) Body weight; (**C**,**D**) representative photographs and terminal tumor weights showing maximal suppression with combination therapy. Tumor volume and weight are expressed as mean ± SD (n = 5 per group). Statistical comparisons were performed using one-way ANOVA with Tukey’s multiple comparison correction. *p* < 0.05 (**) was considered significant. *** indicates *p* < 0.1, * indicates *p* < 0.05.

Finally, we assessed the therapeutic relevance of EZH2 inhibition in vivo. HeLaR xenografts in nude mice were treated intratumorally with Dznep (2 mg/kg) and/or afatinib (12.5 mg/kg) twice weekly for two weeks, and tumor growth was subsequently monitored for a total of three weeks. Tumor growth curves revealed that combination therapy resulted in significantly greater tumor suppression compared with either monotherapy (Figure 6A). No significant body weight loss was observed across treatment groups (Figure 6B). At study endpoint, excised tumors from the combination group were smaller and lighter than those from single-agent or control groups (Figure 6C,D), demonstrating the potential of EZH2 inhibition to overcome afatinib resistance in vivo.

## 4. Discussion

Our study identifies a mechanistic link between radiation-acquired adaptation and reduced sensitivity to EGFR-targeted therapy in cervical cancer. We show that EZH2 overexpression in HeLaR cells suppresses PTEN through promoter methylation, resulting in sustained activation of the PI3K/AKT pathway and decreased responsiveness to afatinib.

Prolonged exposure to radiation can induce durable molecular alterations that enhance survival under subsequent drug treatment [3,4]. Our findings extend this concept by implicating EZH2 as a central epigenetic driver of cross-resistance. Consistent with work in other malignancies, EZH2 can repress PTEN and potentiate oncogenic AKT signaling, thereby conferring treatment tolerance [9,10,12,13]. Reports from prostate cancer suggest polycomb-independent oncogenic functions of EZH2 that converge on AKT activation [10], and similar EZH2-mediated mechanisms have been linked to resistance to PARP inhibitors in ovarian cancer and to EGFR inhibitors in lung cancer [14,15,16,17,18,19,20]. These observations underscore the generalizable role of the EZH2–PTEN–AKT axis in therapy adaptation; our study extends this paradigm to cervical cancer in the context of radiation-acquired change.

EGFR activation drives PI3K/AKT and MAPK/ERK cascades and is associated with poor outcomes and treatment failure [5,6,7]. Head-and-neck studies implicate EGFR signaling in both radio-tolerance and EGFR-TKI insensitivity, yet direct evidence in cervical cancer has been limited. By demonstrating EGFR upregulation alongside reduced afatinib sensitivity in HeLaR cells, our data help bridge this gap.

In vivo, the combination of Dznep and afatinib achieved greater tumor suppression than either agent alone without overt toxicity. Drugs were administered intratumorally, a deliberate choice to maximize local exposure and minimize pharmacokinetic variability, enabling a clearer assessment of tumor-intrinsic responses at this proof-of-principle stage. Future work should explore systemic administration and dose optimization to approximate clinical conditions more closely. From a clinical perspective, current international guidelines for recurrent or radioresistant cervical cancer emphasize systemic chemotherapy, anti-angiogenic therapy with bevacizumab, and immune checkpoint inhibitors as standard treatment options [8]. The incorporation of epigenetic therapies such as EZH2 inhibitors, particularly when combined with EGFR-targeted agents, may complement these established regimens by addressing resistance mechanisms that are not covered by existing standards of care.

Although AKT inhibition (or upstream PI3K blockade) effectively re-sensitized HeLaR cells, ERK inhibition showed limited impact in our assays, suggesting that AKT signaling predominates over ERK in this model. Nonetheless, cross-talk between PI3K/AKT and MAPK/ERK is well documented: reciprocal activation loops, compensatory signaling, and feedback regulation can permit bypass of single-pathway inhibition. Thus, combined targeting of both axes could yield stronger efficacy in heterogeneous tumors and warrants further study.

Limitations should be acknowledged. First, our work focused on a single isogenic pair (HeLa vs. HeLaR), chosen intentionally to mirror the clinical scenario of adaptation after radiotherapy while preserving mechanistic clarity. Validation across additional cervical cancer subtypes and patient-derived models will be important. Second, while we highlight EZH2-mediated PTEN silencing, other resistance mechanisms—such as mutations in EGFR or downstream effectors, epigenetic regulation of additional tumor suppressors, and alternative survival pathways—may contribute. Comprehensive multi-omics profiling coupled with functional perturbation studies will be needed to delineate these complementary mechanisms.

In addition, although our findings based on MSP and ChIP assays strongly support EZH2-mediated epigenetic silencing of PTEN, we acknowledge that bisulfite sequencing would provide higher-resolution validation of promoter methylation. Due to time and resource constraints, this analysis was not feasible within the current study. Future work incorporating bisulfite sequencing or genome-wide methylation profiling will be important to comprehensively confirm the methylation landscape of PTEN in radiation-resistant cervical cancer.

In summary, our results indicate that the EZH2–PTEN–AKT axis is a key driver of reduced EGFR-TKI sensitivity in radiation-adapted cervical cancer cells. Targeting EZH2, potentially in combination with inhibitors of the PI3K/AKT pathway (and, where appropriate, MAPK/ERK), represents a potential avenue to overcome dual resistance phenotypes; further preclinical and clinical studies are warranted to define therapeutic relevance.

## Data Availability

The data used in the review are available upon request.

## Abbreviations

AKT	Protein kinase B
BSA	Bovine serum albumin
ChIP	Chromatin immunoprecipitation
CO_2_	Carbon dioxide
DMSO	Dimethyl sulfoxide
DNA	Deoxyribonucleic acid
Dznep	3-Deazaneplanocin A
ECL	Enhanced chemiluminescence
EGFR	Epidermal growth factor receptor
ERK	Extracellular signal-regulated kinase
EZH2	Enhancer of zeste homolog 2
FBS	Fetal bovine serum
Gy	Gray (unit of radiation dose)
H3K27me3	Trimethylation of histone H3 at lysine 27
HRP	Horseradish peroxidase
IgG	Immunoglobulin G
IRB	Institutional Review Board
MAPK	Mitogen-activated protein kinase
MSP	Methylation-specific polymerase chain reaction
MTS	3-(4,5-dimethylthiazol-2-yl)-5-(3-carboxymethoxyphenyl)-2-(4-sulfophenyl)-2H-tetrazolium assay
OD490	Optical density at 490 nm
PBS	Phosphate-buffered saline
PCR	Polymerase chain reaction
PI3K	Phosphoinositide 3-kinase
PRC2	Polycomb repressive complex 2
PTEN	Phosphatase and tensin homolog
qPCR	Quantitative polymerase chain reaction
RPMI	Roswell Park Memorial Institute medium
SDS-PAGE	Sodium dodecyl sulfate–polyacrylamide gel electrophoresis
SD	Standard deviation
TBST	Tris-buffered saline with Tween 20
TKI	Tyrosine kinase inhibitor
